# Rescue of Epilepsy‐Associated Mutations of the Highly Conserved Glycine Residue 443 in the Human GABA Transporter 1

**DOI:** 10.1096/fj.202403159RR

**Published:** 2025-06-09

**Authors:** Nikita Shah, Vasylyna Kovalchuk, Rocco Zerlotti, Kim Cole, Andre Bazzone, Harald H. Sitte, Thomas Hummel, Ameya S. Kasture, Sonja Sucic

**Affiliations:** ^1^ Institute of Pharmacology, Center of Physiology and Pharmacology Medical University of Vienna Vienna Austria; ^2^ Nanion Technologies GmbH Munich Germany; ^3^ RIGeL Regensburg International Graduate School of Life Sciences Regensburg University Regensburg Germany; ^4^ Hourani Center for Applied Scientific Research Al‐Ahliyya Amman University Amman Jordan; ^5^ Center for Addiction Research and Science Medical University of Vienna Vienna Austria; ^6^ Department of Neuroscience and Developmental Biology University of Vienna Vienna Austria

**Keywords:** 4‐phenylbutyrate (4‐PBA), *Drosophila melanogaster*, epilepsy, pharmacochaperoning, protein folding, small molecules, transporter disease variants, γ‐Aminobutyric acid (GABA) transporter 1 (GAT‐1)

## Abstract

The human γ‐aminobutyric acid (GABA) transporter 1 (hGAT‐1) plays a pivotal role in synaptic neurotransmission by facilitating the clearance of GABA from the synaptic cleft. Pathogenic mutations in the *SLC6A1* gene encoding hGAT‐1 have been implicated in a spectrum of neurodevelopmental disorders, including epilepsy, autism spectrum disorder, intellectual disability, and developmental delay. Here, we elucidate the molecular and functional consequences of disease‐associated mutations affecting the highly conserved glycine residue at position 443 (G443) in hGAT‐1. Through a combination of in vitro biochemical analyses, ion flux assays, and pharmacological profiling in HEK293 cells, alongside in vivo studies in *Drosophila melanogaster* , we demonstrate that substitutions of G443 to aspartate (G443D) or valine (G443V) result in complete abolishment of GABA transport. This severe impairment stems from distinct disruptions in protein folding and trafficking. In particular, G443V is fully retained in the endoplasmic reticulum (ER), as substantiated by de‐glycosylation assays indicating exclusively core‐glycosylated protein bands and confocal co‐localization with the ER chaperone calnexin. The G443D variant, on the other hand, exhibits partial trafficking to the plasma membrane, confirmed by the presence of maturely glycosylated bands, albeit at significantly reduced expression levels relative to the wild type transporter. Treatment with glycerol and 4‐phenylbutyrate (4‐PBA) successfully restored both surface expression and GABA uptake activity of the G443 mutants. Our findings highlight the potential of small‐molecule chaperones as interventions for ameliorating protein misfolding and functional deficits in hGAT‐1‐associated pathologies.

## Graphical abstract

Mutations in the human GABA transporter 1 (hGAT‐1) gene impair GABA transport, leading to developmental disorders like epilepsy and autism. These mutations often disrupt protein folding. Pharmacochaperones can rescue transporter expression and function in heterologous cell lines and in *Drosophila melanogaster* , thus offering potential therapeutic avenues for treating hGAT‐1‐related disorders.

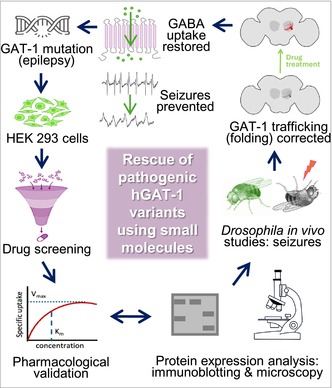

## 1 Introduction

The levels of γ‐aminobutyric acid (GABA) in the human body are regulated by four specific GABA transporters, with GAT‐1 being the chief transporter expressed at the presynaptic nerve terminals. GAT‐1 is the first member of the solute carrier 6 (SLC6) family. It plays an essential role in mediating neurotransmission via rapid reuptake of GABA from the synapse into neurons and astrocytes [1], thus modulating GABAergic signaling that involves the activation of downstream ionotropic and metabotropic GABA receptors. GABA receptor activation takes place in a manner that is precisely synchronized to the timing of GABA release from the presynaptic terminals into extracellular spaces, and to its subsequent clearance from the synaptic cleft [2]. GABA uptake by GAT‐1 is an electrogenic process, which relies on an inward electrochemical gradient, with a co‐transport of sodium and chloride ions through synaptic membranes [2, 3].

The human GAT‐1 (hGAT‐1) is a protein of 599 amino acid residues, arranged into 12 transmembrane (TM) domains, cytoplasmic N‐ and C‐termini, and three consensus N‐linked glycosylation sites in extracellular loop (EL) 2 [4]. Recently published cryogenic electron microscopy (cryo‐EM) structures of GAT‐1 in the inward‐facing state have provided valuable data on the GABA and ion binding pockets [5, 6]. Details of the dynamic structural changes, which take place during substrate translocation, were further refined with AlphaFold2 models of the outward‐facing GAT‐1 conformation [6, 7]. The transport cycle consists of outward‐open, occluded, and inward‐open states—some of which were understood to a degree even prior to cryo‐EM; that is, that conformational transitions are supported by specific domains, for example, glycine 80 in TM 2 confers flexibility to the protein [8, 9]. Certain residues (e.g., tyrosine 60 located in TM 1) are now known to interact with GABA by hydrogen bonding, thus acting as a gate that prevents cytoplasmic substrate release [7].

Modern advances in DNA sequencing have led to the identification of > 200 pathogenic mutations in the gene encoding the human GAT‐1 (hGAT‐1). The reported mutations scatter throughout the transporter [3] and the vast majority are directly linked to neurodevelopmental disorders, including autism and intellectual disability, and a wide spectrum of epilepsy syndromes [10]. Previously, we established that 15 of these variants (e.g., F270S, A288V, A334P, and G550R, among others) exhibit dramatic functional defects (almost complete loss of function) and that the resulting proteins accumulate in the endoplasmic reticulum (ER) to a variable extent, both in transfected HEK293 cells and in transgenic fruit flies [1]. We here probed the molecular features of two recently identified variants associated with childhood epilepsy. The mutations to aspartate (G443D) and valine (G443V) occur at the absolutely conserved glycine residue (G443) in hGAT‐1 (Table 1). G443 is positioned on the fifth extracellular loop (EL 5) of the transporter, juxtamembrane to the TM 10 helix A. The G443D variant (ClinVar RCV001048468) was identified in a 2‐year‐old girl with clinical manifestations of myoclonic atonic epilepsy (MAE, otherwise known as Doose syndrome) and developmental delay [11]. An additional uncharacterized missense mutation, G443V (ClinVar RCV001267259), was originally reported as a variant of uncertain significance (VUS) prone to an inborn genetic disorder phenotype. We showed that point mutations in hGAT‐1 can affect its intracellular trafficking [1]. The resulting GATs can thus be trapped in incorrectly folded states along the folding trajectory in the ER—which is an abode to many molecular chaperones involved in the quality control mechanisms of protein folding [12]. However, this internal molecular chaperone machinery sometimes falls short in assisting the folding intermediates or misfolded proteins in achieving their native folded states. Using heterologous cells and fruit flies, *Drosophila melanogaster*, we here examined the molecular causes via which G443 mutations ultimately lead to seizures in children. Since pharmacochaperoning proved successful in rescuing numerous neurotransmitter transporter variants [13, 14, 15, 16, 17, 18], we screened a collection of small molecules on wild type (WT) and G443 mutant hGAT‐1 proteins. We identified at least two feasible candidate compounds, serving as a proof‐of‐concept for the future design of more effective therapeutic options for this debilitating syndrome.

**TABLE 1:** Sequence alignment of SLC6 transporters indicating the highly conserved glycine residue.

Species	Gene	Name	UniProt ID	Partial sequence
Human	*slc6a1*	GAT1	P30531	CIISYLSNITQG**G** ^**443**^ IYVFKLFDYYSA
	*slc6a2*	NET	P23975	TFSTFLFCITKG**G** ^**465**^ IYVLTLLDTFA—
	*slc6a3*	DAT	Q01959	VLATFLFCVTNG**G** ^**468**^ IYVFTLLDHFA—
	*slc6a4*	SERT	P31645	VITCFLVTLTFG**G** ^**485**^ AYVVKLLEEYA—
	*slc6a5*	GlyT2	Q9Y345	FFIMGFPMITQG**G** ^**625**^ IYMFQLVDTYAA
	*slc6a6*	TAUT	P31641	SYLLGLTMVTEG**G** ^**451**^ MYVFQLFDYYAA
	*slc6a7*	PROT	Q99884	MYLMGLILTTDG**G** ^**446**^ MYWLVLLDDYSA
	*slc6a8*	CRT1	P48029	CFVIDLSMVTDG**G** ^**466**^ MYVFQLFDYYSA
	*slc6a9*	GlyT1	P48067	GFLLGIPLTSQA**G** ^**520**^ IYWLLLMDNYAA
	*slc6a11*	GAT3	P48066	SYFLGLVMLTEG**G** ^**459**^ MYIFQLFDSYAA
	*slc6a12*	BGT1	P48065	CYLIGLFLVTEG**G** ^**444**^ MYIFQLFDYYAS
	*slc6a13*	GAT2	Q9NSD5	SFLVGLIMLTEG**G** ^**439**^ MYVFQLFDYYAA
	*slc6a14*	ATB^0+^	Q9UN76	LFLLGLVCVTQA**G** ^**470**^ IYWVHLIDHFCA
	*slc6a15*	B^0^AT2	Q9H2J7	AFCIGLIFVQRS**G** ^**517**^ NYFVTMFDDYSA
	*slc6a16*	NTT5	Q9GZN6	MFVCGLFFTRPS**G** ^**558**^ SYFIRLLSDYWI
	*slc6a17*	NTT4	Q9H1V8	AFLVGLLFVQRS**G** ^**516**^ NYFVTMFDDYSA
	*slc6a18*	B^0^AT3	Q96N87	CFLSATCFTLQS**G** ^**464**^ NYWLEIFDNFAA
	*slc6a19*	B^0^AT1	Q695T7	TFLIGFIFTLNS**G** ^**478**^ QYWLSLLDSYAG
	*slc6a20*	IMINO	Q9NP91	NCAIGMVFTMEA**G** ^**453**^ NYWFDIFNDYAA
**GABA transporters**				
*C. elegans*	*snf3*	SNF3	G5EBN9	FFCIGIPMVTHS**G** ^**440**^ SHWLTLFDAYGA
Zebrafish	*slc6a1b*	GAT1b	Q5U3E5	SYLIGLSNITQG**G** ^**442**^ LYVFKLFDYYSA
*Drosophila*	*gat*	GAT	Q9V4E7	SYLVGLTCITQG**G** ^**481**^ MYIFQILDSYAV
Rat	*slc6a1*	GAT1	P23978	SYLIGLSNITQG**G** ^**443**^ IYVFKLFDYYSA
Rat	*slc6a13*	GAT2	P31646	SFFIGLIMLTEG**G** ^**439**^ MYVFQLFDYYAA
Rat	*slc6a11*	GAT3	P31647	SYFLGLVMLTEG**G** ^**454**^ MYIFQLFDSYAA
Rat	*slc6a12*	BGT1	P48056	CYLMGLLLVTEG**G** ^**444**^ MYIFQLFDYYAS
Mouse	*slc6a1*	GAT1	P31648	SYLIGLSNITQG**G** ^**443**^ IYVFKLFDYYSA
Mouse	*slc6a13*	GAT2	P31649	SFFIGLIMLTEG**G** ^**439**^ MYVFQLFDYYAA
Mouse	*slc6a11*	GAT3	P31650	SYFLGLVMLTEG**G** ^**454**^ MYIFQLFDSYAA
Mouse	*slc6a12*	BGT1	P31651	CYLMGLLLVTEG**G** ^**444**^ MYIFQLFDYYAS
Bovine	*slc6a1*	GAT1	A0AAF6DMF8	SYLIGLSNITQG**G** ^**450**^ IYVFKLFDYYSA
Bovine	*slc6a6*	TAUT	Q9MZ34	TYLLGLTMVTEG**G** ^**451**^ MYVFQLFDYYAA
Bovine	*slc6a13*	GAT2	A5PJX7	SFLVGLVMLTEG**G** ^**439**^ MYVFQLFDYYAA
**Norepinephrine transporters**				
Rat	*slc6a2*	NET	F1LNR9	TFLLAMFCITKG**G** ^**415**^ IYVLTLLDTFA—
Mouse	*slc6a2*	NET	O55192	TFLLALFCITKG**G** ^**465**^ IYVLTLLDTFA—
**Dopamine transporters**				
Rat	*slc6a3*	DAT	P23977	TFLLSLFCVTNG**G** ^**467**^IYVFTLLDHFA
Mouse	*slc6a3*	DAT	Q61327	TFLLSLFCVTNG**G** ^**467**^ IYVFTLLDHFA—
*Drosophila*	*dat*	DAT	Q7K4Y6	YFVVGLASCTQG**G** ^**467**^ FYFFHLLDRYA—
**Serotonin transporters**				
Rat	*slc6a4*	SERT	P31652	CVLGSLLTLTSG**G** ^**485**^ AYVVTLLEEYA—
Mouse	*slc6a4*	SERT	Q60857	CILGSLLTLTSG**G** ^**485**^ AYVVTLLEEYA—
*Drosophila*	*sert*	SERT	P51905	IFLCALPTMTYG**G** ^**476**^ VVLVNFLNVYG—

*Note*: G, in bold font, at position 443 of hGAT1 and the equivalent glycines, as indicated.

## 2 Material and Methods

### 2.1 Reagents

Cell culture medium, supplements, antibiotics, and other cell culture reagents were acquired from Invitrogen. Sodium dodecyl sulfate (SDS) was obtained from BioMol GmbH (Hamburg, Germany; cat. no. 51430) and 0.4% trypan blue solution was purchased from Sigma–Aldrich (St. Louis, MO, USA; cat. no. T8154‐100ML). Other reagents, such as bovine serum albumin (BSA) and Complete protease inhibitor cocktail (cat. no. 11836170001) were purchased from Roche Applied Science (Mannheim, Germany). [^3^H]GABA (specific activity: 25–40 Ci/mmol; cat. no. NET191250UC) was supplied by Revvity Inc. (Boston, USA). Liothyronine was purchased from Tocris Bioscience (Abingdon United Kingdom; cat. no. 5552). 4‐Phenyl butyric acid (4‐PBA; cat. no. P21005‐25G), pifithrin‐μ (cat. no. 506132) and tiagabine (cat. no. SML0035‐10MG) were all obtained from Sigma‐Aldrich (St. Louis, MO, USA). Guvacine hydrochloride (cat. no. T7557) and 17‐DMAG (cat. no. CAY11036‐5) were purchased from TargetMol Chemicals Inc. (Wellesley Hills, MA, USA) and Cayman Chemical (Michigan, USA), respectively. Noribogaine was from Cfm Oskar Tropitzsch GmbH (Marktredwitz, Germany). Tris was supplied by Carl Roth GmbH (Karlsruhe, Germany).

The primary antibodies, rabbit polyclonal anti‐green fluorescent protein (GFP) (cat. no. ab290) and mouse monoclonal anti‐mCherry (cat. no. ab125096) were purchased from Abcam Plc (Cambridge, UK). The secondary antibodies, goat anti‐rabbit IgG (cat. no. 926–68 071) IRDye 680rd and donkey anti‐mouse IgG (cat. no. 926–32 212) IRDye 800CW were from LI‐COR (Lincoln, Nebraska, USA). All other chemicals were of analytical or reagent grade, obtained commercially. Reagents for solid supported membrane‐based electrophysiology (SSME) buffers were purchased from Carl Roth (Karlsruhe, Germany) and Sigma Aldrich (St. Louis, USA).

### 2.2 Molecular Cloning, Cell Culture and Transfections

The desired mutations of hGAT‐1 were created using the QuikChange Lightning site‐directed mutagenesis kit (Agilent Technologies, Santa Clara, USA; cat. no. cat. no. 210518), using the WT hGAT‐1 (OriGene, cat. no. RC206290) inserted into the pEYFP‐C1 vector (Addgene; cat. no. 6006–1) (YFP‐hGAT‐1) as a template. The mutagenic primers (sequences shown are the sense strands in the 5′ to 3′ direction) were 5′‐AAGAGTTTGAAGACATAAATATCCCCCTGAGTGATGTTAGAG‐3′ for G443D and 5′‐AAGAGTTTGAAGACATAAATAACCCCCTGAGTGATGTTAGAG‐3 for G443V. The mutations were confirmed by automatic DNA sequencing (LGC Labor GmbH Augsburg, Germany).

Human embryonic kidney (HEK293) cells (purchased from the American Type Culture Collection (ATCC); cat. code CRL‐1573) were transiently transfected with the plasmids encoding WT YFP‐hGAT‐1 or the G443V and G443D mutants thereof, using Lipofectamine 2000 (Life Technologies, Carlsbad, USA; cat. no. 11668019) or polyethylenimine (PEI) (23966, Polysciences Europe GmbH, Germany; cat. no. 23966–2). Knock‐down experiments were done using the Ambion Silencer Select siRNAs (Ambion, Carlsbad, USA; cat. no. s6968 and s6969 for HSP70‐1A, s6999 and s7000 for HSP90β and the appropriate negative controls, cat. no. 4390843 and 4390846), using Lipofectamine RNAiMAX (Invitrogen, cat. no. 13778150) for transfections.

In co‐expression experiments, WT YFP‐hGAT‐1 or WT mCherry‐hGAT‐1 were co‐transfected with YFP‐tagged epilepsy variants at different ratios, 1:1 and 1:3 (plasmid ratio by weight), where the amount of WT DNA was maintained constant for all conditions. For imaging experiments examining calnexin overexpression, CFP‐tagged calnexin was added at a quadruple amount relative to the transporters. For uptake assays, 24 h post transfection, the cells were seeded (~10^5^ cells per well) onto poly‐D‐lysine (PDL)‐coated 48‐well plates. For pharmacochaperone experiments, the cells were treated with diverse small molecules for a subsequent 24 h, prior to performing biochemical or functional assays.

### 2.3 Radioligand Substrate Uptake Assays

For uptake assays, the cells were gently washed with 500 μL warm Krebs‐HEPES buffer (KRH) (10 mM HEPES, 120 mM NaCl, 3 mM KCl, 2 mM CaCl_2_, 2 mM MgCl_2_, 2 mM glucose monohydrate, pH 7.3). In pharmacochaperone experiments, the cells were first washed four times with Krebs‐MES buffer (10 mM MES, 120 mM NaCl, 3 mM KCl, 2 mM CaCl_2_, 2 mM MgCl_2_, 2 mM glucose monohydrate, pH 5.5) to remove the tested ligands, prior to the usual KRH buffer washes and commencing uptake. For single concentration point uptake drug screening, the cells were incubated with 50 nM [^3^H]GABA. For saturation uptake kinetics, [^3^H]GABA was diluted with increasing concentrations of unlabeled GABA (1 to 100 μM). Non‐specific uptake (~10% of total uptake for the WT hGAT‐1) was determined in the presence of the GAT‐1‐selective inhibitor tiagabine (10 μM). Uptake was terminated by carefully washing the cells with ice‐cold KRH. The uptake incubation proceeded at room temperature for exactly 3 min. The cells were lysed in 1% SDS, the samples dissolved in scintillation cocktail (Rotiszint eco plus, Carl Roth, Karlsruhe, Germany; cat. no. 0016.3) and radioactive content was measured in a β‐counter.

### 2.4 Confocal Microscopy, Immunocytochemistry and Image Acquisition

HEK293 cells expressing the YFP‐tagged transporters and calnexin‐CFP were seeded onto PDL‐coated 8‐well ibidi glass bottom chambers (ibidi GmbH, Germany; cat. no. 80826). Images were captured using a Zeiss LSM980 inverted microscope with a 63x oil objective. Trypan blue solution (0.05%) was used to stain the plasma membranes. Acquisition settings were kept constant.

For assays in flies, Imaris 9.3 (Oxford instruments) was used to perform volume measurements shown in Figure 6I. Adult fly brains were dissected and fixed in 2% paraformaldehyde (Carl Roth; Cat. no. 0964.1). Subsequently, the brains were washed and blocked in 10% goat serum (Merck; Cat. no. G6767) and incubated with antibodies directed against GFP (1:1000) and neuronal cadherin (1:20, DSHB; cat. no. DN‐Ex #8). Thereafter, the brains were washed and incubated with Alexa Fluor 488 goat anti‐rabbit IgG (1:500; Invitrogen; cat. no. A‐11008) and Alexa Fluor 647 goat anti‐mouse IgG (1:500; Invitrogen; cat. no. A‐21235) secondary antibodies. Images were captured on a confocal microscope. Z‐stack images were scanned at 1.5‐μm section intervals and processed using Image J.

### 2.5 Enzymatic de‐Glycosylation and Immunoblotting

48 h post transfection, the cells were lysed in the NP‐40 buffer, containing 50 mM Tris.HCl (pH 7.4), 150 mM NaCl, 1% Nonidet P‐40 (NP‐40), and 10 mM EDTA supplemented with a protease inhibitor cocktail (Roche Complete; cat. no. 11836170001). The lysates were incubated by rotation at 4°C for 1 h and then centrifuged (40 min at 16000 g at 4°C) to remove any insoluble material. 20 μg of total protein was incubated in the absence or presence of endoglycosidase H (EndoH) (New England Biolabs, MA, USA; cat. no. P0702S) for 2 h at 37°C according to the manufacturer's instructions. For thermal stability experiments, lysates were either incubated at room temperature (i.e., non‐heated controls) or heated (at increasing temperatures for 15 min). The proteins were resolved on SDS denaturing polyacrylamide gels (resolving gel: 10%) by electrophoresis and transferred onto polyvinylidene difluoride (PVDF) membranes. The membranes were blocked in a solution containing 5% non‐fat skimmed milk in Tris‐buffered saline containing 0.1% Tween 20 (TBST) for 1 h at room temperature, and then incubated with a rabbit polyclonal anti‐GFP antibody (1:5000 dilution) overnight at 4°C. For co‐expression immunoblotting experiments, a mouse monoclonal anti‐mCherry antibody was used additionally (1:5000 dilution). An antibody against G protein β (1:1000 dilution) was used to ensure equal amounts of protein loading. Immunoreactivity was detected by fluorescence on Odyssey CLx (9140, LI‐COR, Lincoln, Nebraska USA), using the goat anti‐rabbit secondary antibody (1:5000 dilution) (IRDye 680rd, LICOR) and donkey anti‐mouse secondary antibody (1:5000 dilution) (IRDye 800CW, LICOR).

### 2.6 Membrane Vesicles and SSME Sensor Preparation From HEK293 Cells

Membrane vesicles from HEK293 cells overexpressing WT hGAT‐1 and G443D were obtained as reported in Bazzone et al., 2022 [19]. The purified vesicles were then rapidly frozen in liquid nitrogen and stored at −80°C, then thawed prior to SSME sensor preparation. The sensors were prepared as previously described [20] in resting buffer (R buffer, 30 mM HEPES, 5 mM MgCl2, 140 mM KCl, titrated to pH 7.4 with NMDG^+^). The sample total protein concentration, measured via Bradford assay, was 1.8 mg/mL for WT hGAT‐1 vesicles and 1.5 mg/mL for G443D. Both samples were diluted 10‐fold in R buffer before being added to the SSME sensors.

### 2.7 SSME Measurements

SSME recordings were conducted using a SURFE^2^R N1 device (Nanion Technologies GmbH) operating with the SURFE^2^R N1 Control 1.7.0.2 software, as previously described [20, 21]. In brief, the device performs a rapid solution exchange between R, NA, and A solutions, while transport is driven exclusively by substrate (and co‐substrate) concentration gradients, as no controlled membrane voltage is applicable in SSME. Non‐activating buffer (NA buffer) composition was 30 mM HEPES, 5 mM MgCl_2_, 140 mM NaCl, titrated to pH 7.4 with NMDG^+^. A buffer composition was 30 mM HEPES, 5 mM MgCl_2_, 140 mM NaCl, titrated to pH 7.4 with NMDG,^+^ and GABA was added at different concentrations. The experimental protocol consisted of a single 1 s rinse in R buffer, followed by sequential solution perfusion steps: 1 s perfusion with NA buffer, 1 s perfusion with activating buffer (A buffer), and a final 1 s perfusion with NA buffer. Solutions were perfused at a flow rate of 200 μL/s, corresponding to total volumes of 1 mL R buffer, 0.6 mL NA buffer, and 0.3 mL A buffer per measurement. The currents were recorded at a sampling rate of 1 kHz. All measurements were performed at room temperature (23 ± 1)°C.

### 2.8 *Drosophila* Genetics, Drug Treatment and Heat‐Induced Seizure Assay

The transgenic reporter lines for YFP–tagged hGAT‐1 G443V and G443D were generated using the pUASg attB vector (gift from Dr. Bischof and Dr. Basler, University of Zurich) and injected into embryos of ZH‐86Fb flies (Bloomington stock no. (BDSC) 24 749). The transgenic reporter line for WT YFP‐hGAT‐1^1^ was used to express tagged‐hGAT‐1. All flies were kept at 25°C in a 12‐h light/12‐h dark cycle, and the crosses were performed at 25°C. ALRM Gal4 (BDSC 67031), SS02766 Gal4 (GABAergic ellipsoid body neurons, BDSC 75994), GAD‐1 Gal4 (BDSC 51630), UAS‐mCD8‐GFP (FBti0012686), UAS‐KDEL‐RFP (BDSC 30909) were ordered from the Bloomington Drosophila stock center (Bloomington, IN). The ALRM (astrocytic leucine‐rich repeat molecule) Gal4 driver line was used to drive gene expression in astrocytes. The GAD‐1 (Glutamic Acid Decarboxylase 1) and SS02766 (GABAergic ellipsoid body neurons) driver lines were used to drive expression in GABAergic neurons. To visualize cellular structures, the UAS‐mCD8‐GFP reporter line (murine CD8a fused to GFP) was used to label neuronal and astrocytic membranes, while the UAS‐KDEL‐RFP reporter line (RFP fused to an ER retention sequence, KDEL) was used to label the ER compartment. The flies received standard cornmeal food, supplemented with water, 1 mM 4‐PBA, or 10% glycerol. For seizure assay, at least ten 3–5‐day‐old male flies of said genotypes were immersed in a water‐bath at 40°C for a period of 1–5 min. Fruit flies exhibit a distinct repertoire of seizure‐like behaviors upon mechanical or thermal stimulation [22, 23]. Seizure‐like episodes were defined as an initial period of brief leg twitches and wing flapping, followed by failure to maintain a standing posture that leads to a paralysis state. This phase is followed by a recovery phase, during which flies attempt to regain posture, manifested by wing flapping and leg twitching. The flies in due course recover the normal posture.

### 2.9 *In Silico* Analysis

ColabFold v1.5.5 software [24] was used to predict the structural alterations induced by the G443D and G443V mutations. The generated PDB files were analyzed using ChimeraX 1.9 [25]. AlphaFold PDB P30531 served as a control in the structural analyses. hGAT‐1 has disordered regions in its cytoplasmic amino (N) and carboxyl (C) termini. The ColabFold v1.5.5 software predicts per‐residue confidence metrics (pLDDT), with low values estimated for cytoplasmic domains. The remaining hGAT‐1 structure showed high pLDDT values, with the exception of residues 358 to 363 (https://alphafold.ebi.ac.uk/entry/P30531).

## 3 Results

### 3.1 In Vitro Characterization of Epilepsy‐Triggering Mutations at Residue G443 of hGAT‐1

**FIGURE 1:**
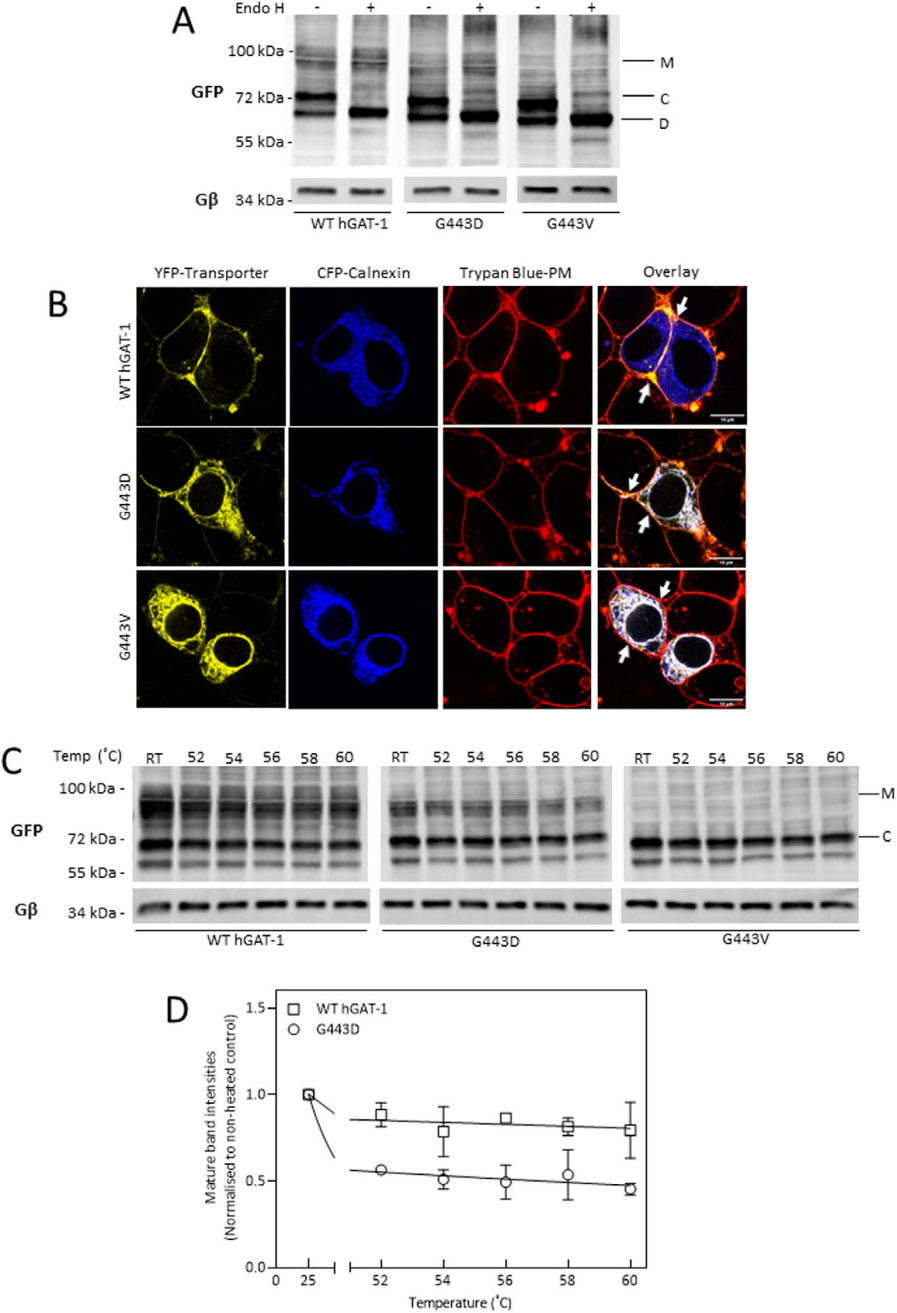
In vitro characterization of epilepsy variants of residue G443 in hGAT‐1. (A) De‐glycosylation profiles of the WT hGAT‐1 and the mutants were studied by endoglycosidase (Endo) H and immunoblotting, as described in Methods. M, C, and D signify mature glycosylated, core‐glycosylated, and de‐glycosylated bands, respectively. (B) HEK293 cells expressing the N‐terminal YFP‐tagged GATs were visualized by confocal microscopy, using trypan blue to delineate the plasma membrane and calnexin‐CFP to label the ER compartment (scale bars represent 10 μm). The WT hGAT‐1 was properly targeted to the cell surface, unlike the mutants, which indicated full to partial intracellular expression. The expression pattern of the G443 mutants (panel A) was consistent with the confocal images, attesting to distinct degrees of ER‐retention. (C) Representative immunoblots showing the thermal stability of WT hGAT‐1 and the G443 mutants. (D) Thermal shift curves of data quantified from at least three independent assays, with mature band intensities normalized to their pertinent controls (error bars = S.D).

We first examined the endoglycosidase (Endo) H de‐glycosylation profiles of WT hGAT‐1 and mutants thereof. ER‐resident proteins are core‐glycosylated and hence sensitive to de‐glycosylation by Endo H. Plasma membrane‐bound proteins are maturely modified with multiple glycosylations at the Golgi cisternae and are resistant to Endo H. Accordingly, in Figure 1A, two protein bands were detected for the WT hGAT‐1; lower band species were sensitive to enzymatic digestion (and de‐glycosylation) by Endo H, that is, thus representing the core‐glycosylated ER‐retained proteins. The upper band, resistant to the enzyme, demonstrated the mature glycosylated species. In contrast, the G443V variant presented only core‐glycosylated (i.e., ER‐stalled) species, whereas G443D exhibited some mature glycosylated (plasma membrane‐bound) bands (Figure 1A). To further validate these results, we studied the cellular distribution patterns of all three transporters using confocal laser scanning microscopy: the images indicated that both G443V and G443D differ in their expression from the WT hGAT‐1 (Figure 1B, YFP‐channel images). Cyan fluorescent protein (CFP)‐tagged calnexin was used to mark the ER regions and trypan blue to delineate the plasma membranes of cells. Although a fraction of the G443D variant was trapped within the cells, it also bore substantial co‐localization with the plasma membrane (middle panel in Figure 1B, white arrows). Surprisingly, the G443V protein showed complete retention in the ER, manifested by its co‐localization with the ER‐resident chaperone calnexin (Figure 1B, bottom panel). We hence inferred that mutations of G443 give rise to partially‐ to completely misfolded GAT proteins. Further, we examined the thermal stability of both mutants, compared to the WT hGAT‐1. The mature bands of G443D exhibited reduced thermal stability with increasing temperatures (middle panel in Figure 1C) relative to the WT (left panel, Figure 1C), whereas G443V could not be detected due to its absence from the plasmalemma (Figure 1C, right panel). Mature band intensities were quantified relative to non‐heated controls for the WT hGAT‐1 and G443D (Figure 1D).

**FIGURE 2:**
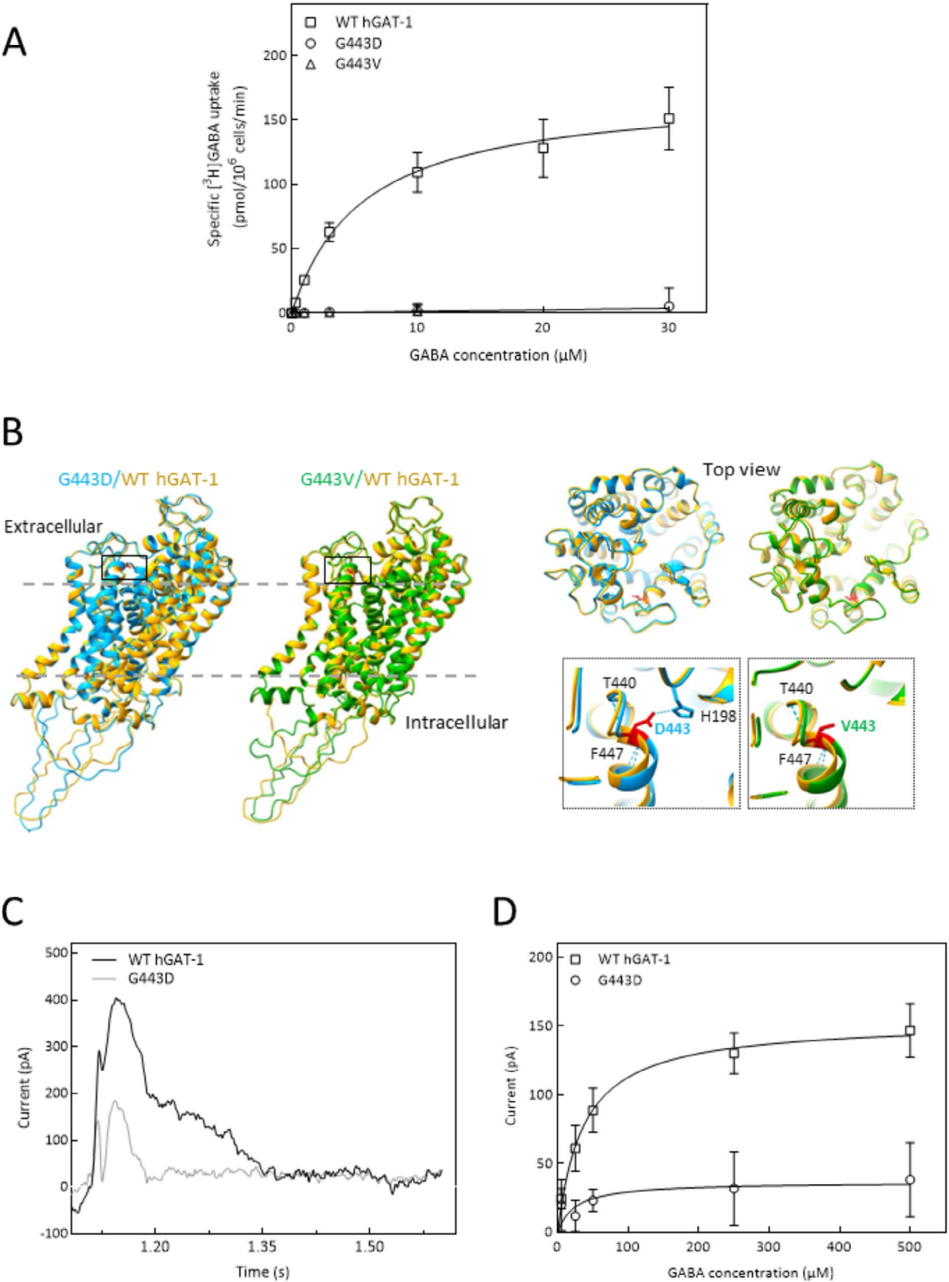
Structural and functional analysis of G443 hGAT‐1 variants. (A) HEK293 cells were transiently transfected with the cDNA encoding YFP‐tagged WT hGAT‐1 or the mutants, G443D and G443V, to perform Michaelis–Menten uptake kinetics. Specific [^3^H]GABA uptake was measured for exactly 3 min, as described in detail under Methods. Non‐specific uptake was determined in the presence of 10 μM tiagabine. The data were obtained from at least three independent experiments, performed in triplicate (error bars = S.D.). Both mutants displayed a loss‐of‐function phenotype. (B) Protein structure predictions were generated using ColabFold v1.5.5 software [24]. The computed ribbon structures of G443D (shown in blue) and G443V (shown in green) were compared to the WT hGAT‐1 (shown in yellow). Residue 443 is indicated in bright red sticks. The top view displays the extracellular loops of superimposed transporters. Dotted squares show magnified views of residue 443, with the superimposition of G443D (blue) and G443V (green) onto WT hGAT‐1. Blue dotted lines indicate hydrogen bonds. (C) Averaged traces obtained with SSME recordings on hGAT‐1 WT (black traces) and G443D (gray traces). The measurements were performed on three sensors each for WT and G443D. The sensors were prepared in R buffer, then the NA solution was perfused to generate the 140 mM Na + inward‐directed gradient, and finally the A buffer with 1 mM GABA activated hGAT‐1 transport. (D) Amplitude of currents at 0.17 s after GABA perfusion for both hGAT‐1 WT and hGAT‐1 G443D. Different A buffers were perfused with GABA concentrations from 5 μM to 500 μM, at one concentration per sensor. The data averages over three sensors for each concentration point, for a total of 15 sensors used for each, WT and G443D. The plotted data points (error bars = S.D.) were fitted to a Michaelis–Menten function.

Upon assessing the activity of WT hGAT‐1 and the G443V and G443D variants in Michaelis–Menten GABA uptake kinetics assays, we found that neither mutant had appreciable GABA uptake (Figure 2A), as opposed to the saturable uptake by the WT transporter (i.e., K_m_ = 5.6 ± 0.8 μM and V_max_ = 172 ± 7.1 pmol.10^−6^cells.min^−1^). Evidently, this glycine residue plays a crucial functional role. As shown in Table 1, G443 of hGAT‐1 is strictly conserved across species and all members of the SLC6 family. It is situated at the start of an alpha helix and is tightly packed above the transmembrane region (Figure 2B). Protein structure predictions for G443D and G443V show that these mutations affect the packing of extracellular loops (Figure 2B). However, they did not affect membrane‐embedded regions. Cytoplasmic N‐ and C‐termini harbor disordered segments, and hence the model confidence for these regions (shown as ribbon structures in Figure 2B) is low. To assess the impact of the mutations on protein stability, we used three support vector machine (SVM)‐based algorithms to calculate ΔΔG values [26, 27, 28], which predicted that both G443D and G443V decrease GAT‐1 stability (Table 2).

**TABLE 2:** Protein stability assessment using *in silico* approaches.

Mutation	MUpro	I‐Mutant v2.0	INPS‐MD
	ΔΔG value	Stability	ΔΔG value
**G443D**	−0.189 (decreased stability)	Reduced	−1.88 (destabilizing)
**G443V**	−0.05 (decreased stability)	Reduced	−1.27 (destabilizing)

Upon applying 1 mM GABA in the presence of a 140 mM Na^+^ inward‐directed gradient to HEK293 membrane vesicles in SSME, WT hGAT‐1 originated a complex current with three distinct phases (Figure 2C, black trace). When multi‐phasic currents are detected in SSME, the fast‐decaying components (pre‐steady‐state currents or PSS) are usually associated with rapid conformational rearrangements of the protein upon substrate binding, while the slower‐decaying component is usually attributed to the steady‐state transport reaction [19, 29, 30, 31, 32]. The G443D mutant exhibited the first two phases, albeit significantly reduced in amplitude, while the third phase was entirely absent (Figure 2C, gray trace). Reading the current amplitude at 0.17 s after GABA perfusion—the slowest‐decaying phase corresponding to transport—we observed a saturable behavior (with a K_m_ of 36 ± 7.7 μM and V_max_ of 153.2 ± 8.1 pA), while no significant transport was measured for the G443D mutant (Figure 2D). These results are in agreement with the absence of radioligand GABA uptake in G443D, described above (Figure 2A). In addition, SSME revealed that substrate binding and the associated conformational rearrangements appear to be impaired in G443D; that is, PSS currents showed a significantly lower amplitude compared to WT while applying the same GABA concentration.

### 3.2 Rescue of G443 Variants by Pharmacochaperoning In Vitro

**FIGURE 3:**
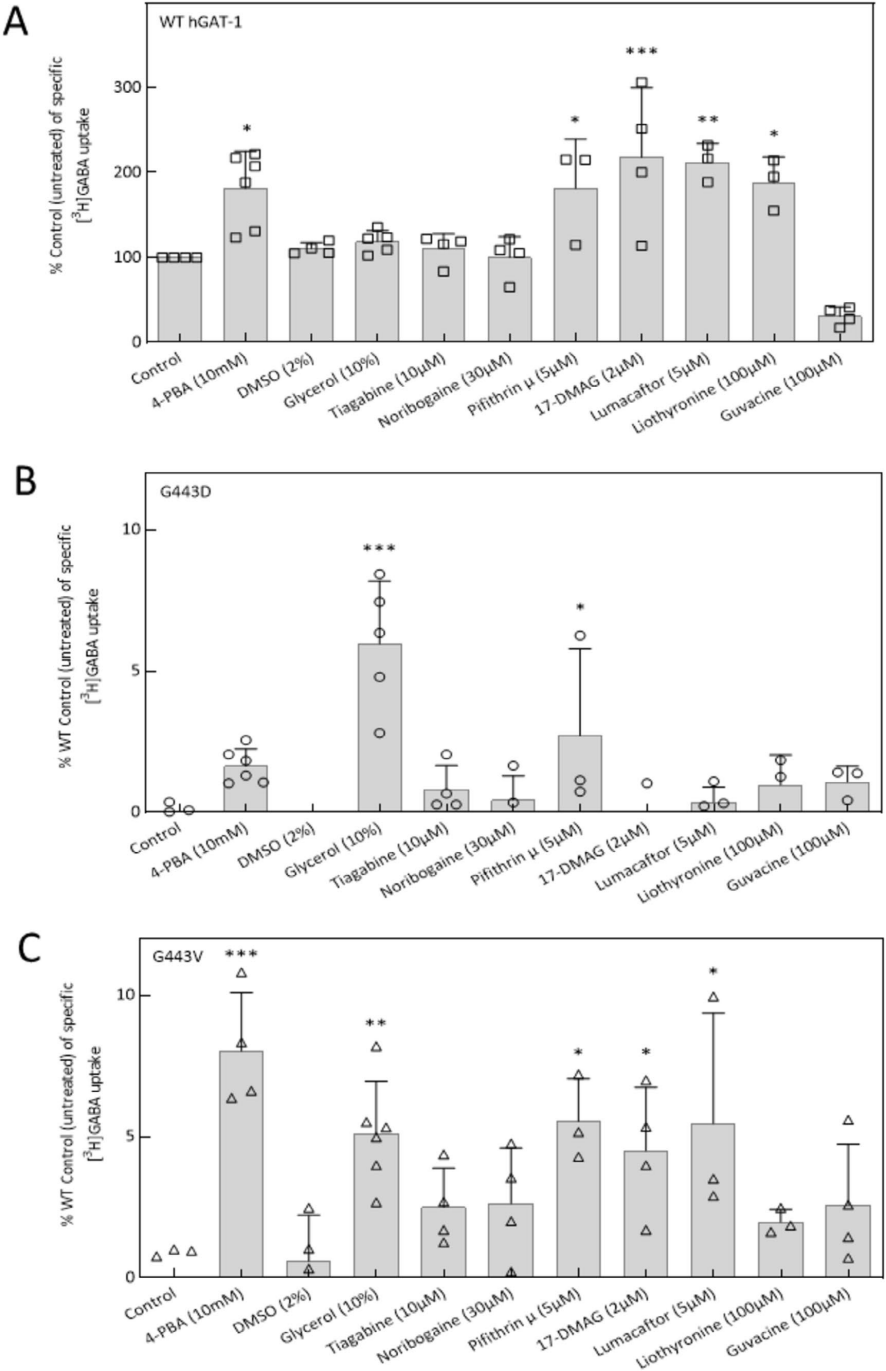
Small molecule in vitro screening for their rescue potential on G443 variants. (A, B and C) HEK293 cells were transiently transfected with plasmids driving the expression of YFP‐tagged WT hGAT‐1 and mutants. After 24 h, the cells were seeded onto PDL‐coated 48‐well plates and incubated in the absence or presence of various drugs (i.e., 4‐PBA (10 mM), DMSO (2%), glycerol (10%), tiagabine (10 μM), noribogaine (30 μM), pifithrin‐μ (5 μM), 17‐DMAG (2 μM), lumacaftor (5 μM), liothyronine (100 μM) and guvacine (100 μM)) for a subsequent 24 h. Single concentration [^3^H]GABA uptake assays were performed, as described under Methods. Glycerol (10%) and 4‐PBA (10 mM) were identified as beneficial candidate compounds for G443D and G443V, respectively. The data were obtained from at least three independent experiments performed in triplicate (error bars represent S.D.). All values were normalized to WT hGAT‐1 control. The data sets were statistically compared by one‐way ANOVA, followed by Tukey's post hoc *t*‐tests (*p < 0.05, **p < 0.01, ***p < 0.001).

Misfolded SLC6 proteins are amenable to rescue upon exposure to certain small molecules, known as chemical and pharmacological chaperones. Chemical chaperones are low‐molecular‐weight compounds, which non‐selectively interact with their client proteins and in turn support one or more processes, from folding and precluding aggregation to reducing ER and cellular stress [12, 33, 34, 35]. Pharmacological chaperones, on the other hand, selectively bind to target proteins and stabilize them [13, 14, 33, 36, 37, 38]. Pharmacochaperoning rectifies folding defects in impaired mutant transporters, restoring their expression and functionality [33, 34]. In our recent studies, 4‐PBA rescued several hGAT‐1 variants in vitro and in vivo [1]. However, in the present study, 4‐PBA failed to mend the expression or activity of the partially misfolded, loss‐of‐function G443D mutant (Figure 3B, *second bar*). The fully misfolded G443V variant, on the other hand, was amenable to 4‐PBA‐induced rescue (Figure 3C, *second bar*) in transiently transfected HEK293 cells. We also assessed the chaperone capacity of glycerol. Glycerol treatment is known to enhance the expression of misfolded mutants, as observed with the ΔF508 mutation of the cystic fibrosis transmembrane conductance regulator (CFTR) [39]. In our experiments on the G443D variant—which was irresponsive to 4‐PBA—we observed promising effects following a 24‐h exposure to glycerol (Figure 3B, *fourth bar*).

Several small molecules exerted positive effects on WT hGAT‐1, enhancing GABA uptake (Figure 3A). Chaperones are presumed to orchestrate multiple cellular mechanisms to facilitate the proper trafficking of target proteins, directing them to their designated sites, such as the plasma membrane for GAT‐1 and other SLC6 transporters [3]. Here, we also modulated the heat shock protein (HSP) relay in HEK293 cells through pharmacological inhibition or siRNA‐induced knockdown. These approaches are believed to relax the stringent quality control of protein folding in the ER and have previously been shown to enhance the cell surface expression of serotonin transporter (SERT) [17, 37, 38], dopamine transporter (DAT) [13, 14] and creatine transporter 1 (CRT‐1) [40, 41] mutants. However, siRNA knockdown produced only modest effects on G443D and G443V (Figure S1). While inhibition of HSPs 70 and 90 led to partial rescue of the G443V variant (Figure 3C), we did not pursue further studies with these compounds due to their cytotoxic effects.

**FIGURE 4:**
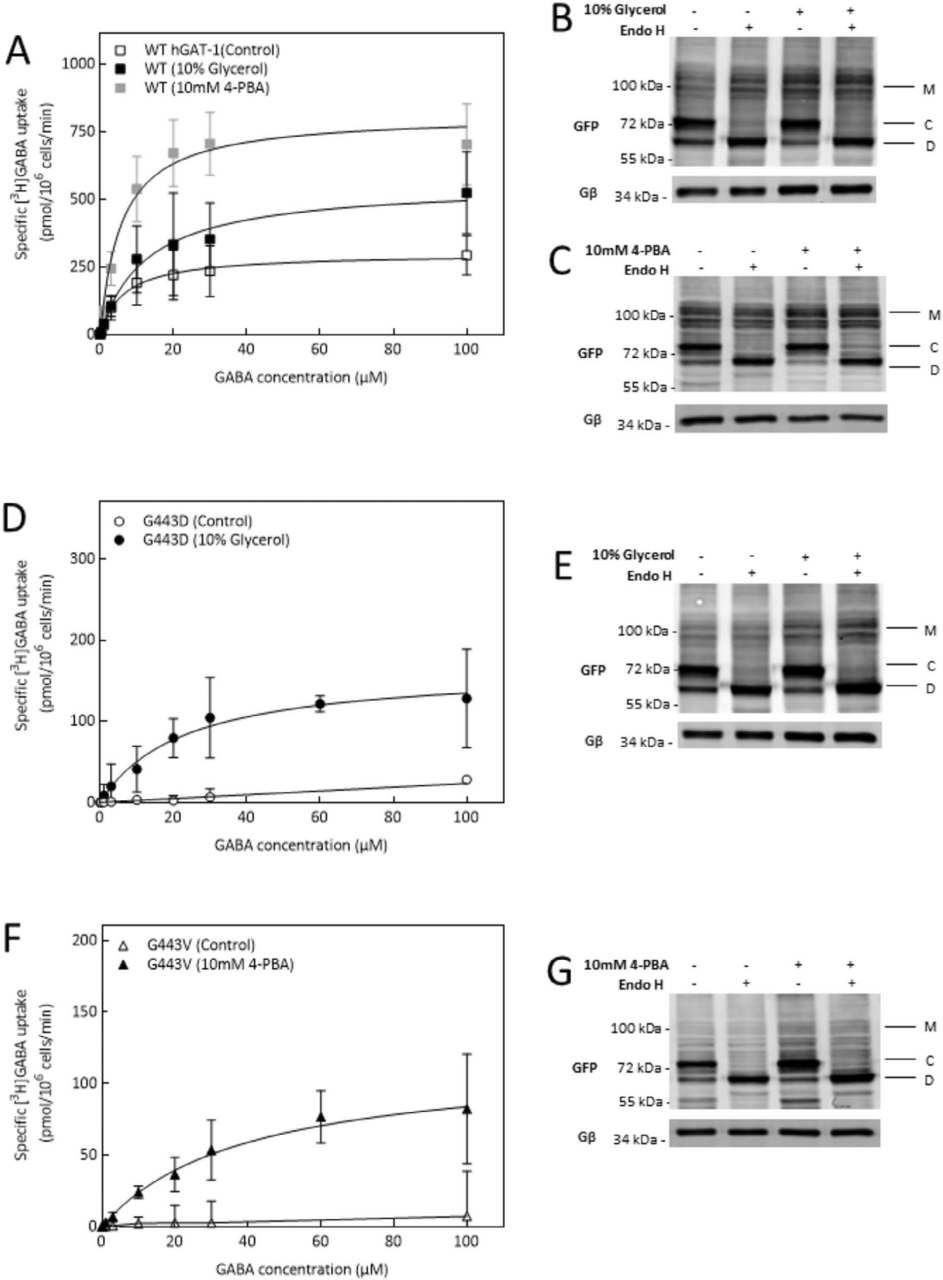
Functional and biochemical validation of G443D and G443V rescue by chemical chaperones in vitro. (A) Michaelis–Menten [^3^H]GABA uptake kinetics of WT hGAT‐1 was carried out in transiently transfected HEK293 cells, with and without 24 h treatment with glycerol (10%) and 4‐PBA (10 mM). The kinetic parameters, K_m_ and V_max_ values, were calculated from mean data measured for each drug condition. (B and C) Representative blot images before and after treatment of WT hGAT‐1 with glycerol and 4‐PBA, respectively. (D) Michaelis–Menten [^3^H]GABA uptake kinetics of the G443D mutant was measured in the absence or presence of glycerol (10%). Panel (E) immunoblot reveals enhanced surface expression after glycerol treatment. (F) HEK293 transiently transfected G443V were treated with or without 4‐PBA (10 mM), prior to measuring uptake kinetics. Panel (G) is a representative blot showing the rescue of mature cell surface bands after 4‐PBA treatment. GABA uptake data were acquired from at least three independent experiments performed in triplicate (error bars denote S.D.).

In short, we screened a compendium of small molecules, from GAT‐1 ligands (i.e., tiagabine, guvacine and liothyronine) and known pharmacochaperones of monoamine transporters (i.e., noribogaine) to non‐specific chemical chaperones (i.e., glycerol, dimethyl sulfoxide (DMSO) and 4‐PBA) and HSP70 and 90 blockers (i.e., pifithrin‐μ and 17‐DMAG, respectively). Given that glycerol and 4‐PBA were the most effective molecules in rescuing G443D and G443V, respectively (Figure 3), their effects were further investigated through Michaelis–Menten GABA uptake kinetics (Figure 4; WT hGAT‐1, control: K_m_ = 6.5 ± 1.1 μM, V_max_ = 298.2 ± 14.4 pmol.10^−6^cells.min^−1^; glycerol: K_m_ = 12.7 ± 4.1 μM, V_max_ = 556.4 ± 63.6 pmol.10^−6^cells.min^−1^ and 4‐PBA: K_m_ = 5.7 ± 0.8 μM, V_max_ = 811.6 ± 30.8 pmol.10^−6^cells.min^−1^). The kinetic parameters indicated that glycerol and 4‐PBA recovered saturable GABA uptake activity by G443D (K_m_ = 22.9 ± 6.7 μM, V_max_ = 165.3 ± 20.3 pmol.10^−6^cells.min^−1^) and G443V (K_m_ = 39.2 ± 9.7 μM, V_max_ = 116.5 ± 13.5 pmol.10^−6^cells.min^−1^), respectively. We validated these functional data by examining the de‐glycosylation profiles of WT and the variants (Figure 4). Their cell surface expression after pharmacochaperoning could be detected as mature glycosylated protein species (Figure 4). Accordingly, G443D presented higher mature glycosylation after glycerol treatment (‘M' bands in Figure 4E). Remarkably, G443V, which was totally omitted from the plasma membrane in the absence of treatment, produced mature glycosylated bands upon exposure to 4‐PBA (‘M' bands in Figure 4G). Above all, chaperone treatment drastically augmented the mature glycosylated bands of WT hGAT‐1 (Figure 4B,C). Moreover, we investigated whether G443 mutations influenced the trafficking of WT hGAT‐1 when co‐expressed in HEK293 cells (Figure S2). Co‐expression of WT with G443V or A288V at a 1:1 ratio reduced WT uptake by approximately 30%–40%, whereas G443D had no effect on WT activity. At a 1:3 ratio, WT uptake was markedly reduced for all three mutants (Figure S2A). Immunoblots from co‐transfections using distinct fluorescent tags—mCherry‐WT hGAT‐1 (red channel) and YFP‐mutants (green channel)—corroborated these findings, aligning with the uptake data. The mature glycosylated bands of WT co‐expressed with G443D at a 1:1 ratio resembled those of WT transfected with the empty vector (control). In contrast, co‐expression with G443V at the same ratio led to a reduction in the surface‐bound (mature) species of WT (Supplementary Figure S2B, quantified in panel C). To visualize whether the mutants interfere with WT hGAT‐1 trafficking, we performed confocal imaging (Figure S3A). WT hGAT‐1 was correctly targeted to the plasma membrane when co‐transfected with G443D (at a 1:1 ratio), whereas G443V caused partial intracellular retention of WT. A similar phenomenon was previously reported for a glycine transporter 2 (GlyT2) mutation linked to hyperekplexia, where the mutant trapped WT intracellularly—a defect that was corrected by overexpressing calnexin [42]. Following this approach, we overexpressed calnexin at a 4:1 ratio relative to transporter cDNA. Calnexin overexpression had no effect on WT trafficking when co‐expressed with G443D. However, when co‐expressed with G443V, it rescued the ER‐trapped WT species, restoring proper plasma membrane delivery (Figure S3B).

### 3.3 In Vivo Studies on G443 Variants Using *Drosophila* as an Animal Model

**FIGURE 5:**
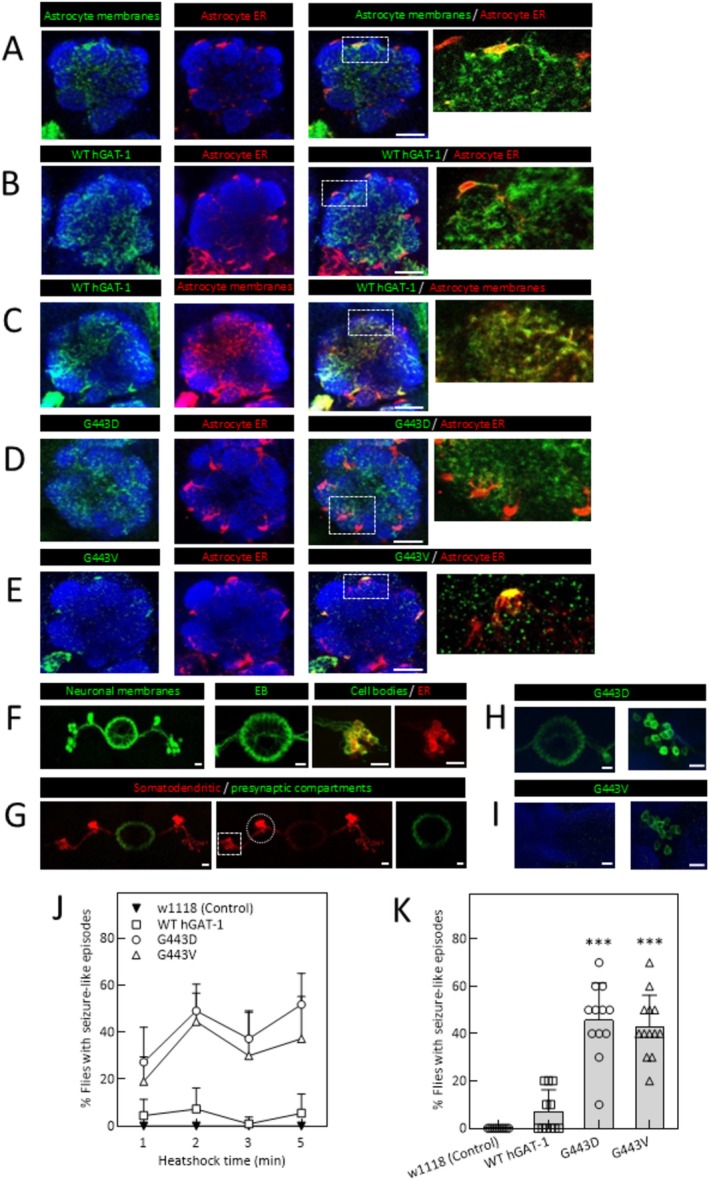
The expression pattern of G443D and G443V epilepsy variants in adult fly brains. (A) Visualization of glial membrane (green) and ER (red) in the AL neuropile. The magnified view (dotted square) denotes a merged image, where the glial ER is limited to the cell bodies, while the membranes cover the larger portion in the AL neuropile region. (B) Co‐expression of WT hGAT‐1 (green) and glial ER (red) in the astrocytes of adult fly brains. The magnified view (dotted square) shows that the distribution of the WT hGAT‐1 differs from the glial ER marker. (C) Co‐localization of WT hGAT‐1 (green) and membrane marker (red). The magnified view (dotted square) shows the co‐expression of WT hGAT‐1 and the membrane marker in glial processes. (D) and (E) show co‐expression of G443D (panel D, green) and G443V (panel E, green) with the glial ER marker (red). The magnified view denotes that G443D has a widespread distribution in the glial processes (D), while G443V is restricted to the ER compartment in astrocytes (E). (F) Visualization of membrane (green) and ER compartments (red) in GABAergic EB neurons. The ER compartment is restricted to the cell bodies. (G) Somatodendritic (red, dotted square shows cell bodies, dotted circle shows dendritic region) and presynaptic (green) compartments of the GABAergic EB neurons. (H) G443D mutant shows robust presynaptic enrichment and expression in the GABAergic neurons. (I) G443V mutant mimics the ER‐targeted expression and is absent from the presynaptic region. Scale bar (A to E) = 20 μm and (F to I) = 10 μm. (J) and (K) Seizure susceptibility in different genotypes is shown as the percentage of flies with seizure‐like episodes, after heat induction at 40°C. Ten three‐to‐five‐day‐old male flies of genotypes WT hGAT‐1 (ALRM Gal4; UAS‐YFP WT‐hGAT‐1), G443D (ALRM Gal4; UAS‐YFP hGAT‐1‐G443D), G443V (ALRM Gal4; UAS‐YFP hGAT‐1‐G443V) and w1118 were immersed in a water‐bath for 1, 2, 3, and 5 min, and seizure activity was recorded (J). The percent of flies with seizure‐like episodes after 2 min of water‐bath immersion was plotted and used for further analysis (K). Genotypes: (A) UAS‐KDEL‐RFP/ALRM Gal4; UAS‐mCD8‐GFP/+; (B) UAS‐KDEL‐RFP/ALRM Gal4; UAS‐YFP‐ WT hGAT‐1; (C) ALRM Gal4/+; UAS‐YFP WT‐hGAT‐1/UAS‐mCD8‐RFP; (D) UAS‐KDEL‐RFP/ALRM Gal4; UAS‐YFP hGAT‐1‐G443D/+; (E) UAS‐KDEL‐RFP/ALRM Gal4; UAS‐YFP‐hGAT‐1‐G443V/+; (F) UAS‐KDEL‐RFP/R20D01‐p65.AD; UAS‐mCD8‐GFP/R72B07‐GAL4.DBD; (G) UAS‐DenMark, UAS‐Syt‐GFP; R20D01‐p65.AD; R72B07‐GAL4.DBD/+; (H) R20D01‐p65.AD/+; UAS‐YFP hGAT‐1‐G443D/R72B07‐GAL4.DBD; (I) R20D01‐p65.AD/+; UAS‐YFP hGAT‐1‐G443V/R72B07‐GAL4.DBD. Each image is representative of at least 10 additional images per condition. Neuropiles are labeled by NCAD in blue. The statistical comparison was done by one‐way ANOVA followed by Dunnett's test (***p < 0.001).

We previously studied one of the most recurrent hGAT‐1 epilepsy variants, A288V, in *Drosophila melanogaster* [1]. We assessed both astrocytic and neuronal expression of the resulting protein, which was retained in the ER [1]. In the current study, we aimed to investigate the impact of G443 mutations on GAT‐1 protein folding and trafficking pathways in an animal model. To this end, we generated transgenic flies expressing fluorescently labeled hGAT‐1 G443D and G443V variants. In the antennal lobe (AL) of the adult *Drosophila* brain, astrocytes are located at the periphery of the neuropile and extend cellular processes into the central area of synaptic glomeruli [43, 44]. Typically, astrocytes are widely expressed in the antennal lobe (AL) of the adult fly brain. We visualized the membranes and the ER of astrocytes in a single section of the AL (Figure 5A) using an astrocyte‐specific ALRM Gal4 driver line [45]. When we co‐expressed the YFP‐tagged WT hGAT‐1 [1], along with the markers of the ER (Figure 5B) and membrane regions (Figure 5C), we found that the WT hGAT‐1 was abundantly expressed, strongly resembling the pattern presented by the membrane marker. Similar to the WT, the G443D mutant bore a widespread expression at the membrane region (Figure 5D), while G443V is highly enriched in the astrocyte cell body and co‐localizes with the ER (Figure 5E).

We probed the neuronal expression of both mutants in a subset of ellipsoid body (EB) neurons, identified as being GABAergic in nature (Figure 5F) [46]. We visualized the neurons by co‐expressing membrane and ER markers (Figure 5F). We also defined the somatodendritic (Figure 5G, shown in red) and presynaptic compartments (Figure 5G, shown in green) of EB neurons to elucidate whether the G443D mutant can traffic to its proper presynaptic sites and whether variant G443V resides in the cell bodies—which was the case. As anticipated, G443D exhibited presynaptic enrichment (Figure 5H), in contrast to the ER‐stalled phenotype of G443V (Figure 5I). Similar patterns were observed with broader neuronal expression of the two mutants (Supplementary Figure S4).

In our prior investigations, we explored whether mechanical stress (bang‐sensitive [47]) and heat stress (heat‐induced) could induce a seizure phenotype in flies expressing the A288V mutant, one of the most recurrent mutations in the *SLC6A1* gene [1]. We discovered that heat stress triggered seizures in A288V flies, while mechanical stress failed to elicit a similar response. To assess the potential seizure phenotypes of G443D and G443V, vials containing three‐to‐five‐day‐old male flies expressing these variants, along with WT controls, were subjected to a 40°C water bath for a duration of up to 5 min. The flies were monitored for a divergent behavioral repertoire [22]. They displayed initial seizure‐like activity (characterized by wing flapping and leg twitching), an ensuing inability to maintain posture, and the consequent paralysis phase. This was followed by wing flapping and leg twitching and, eventually, normal posture recovery. The seizure‐like repertoire was observed for both mutants as early as 30 s after immersion into the water‐bath. A little over 40% of flies exhibited seizure susceptibility for both G443D and G443V after a 2 min immersion in water (*p* = < 0.001, Figure 5J,K). We observed no locomotor defects in either mutant (data not shown).

**FIGURE 6:**
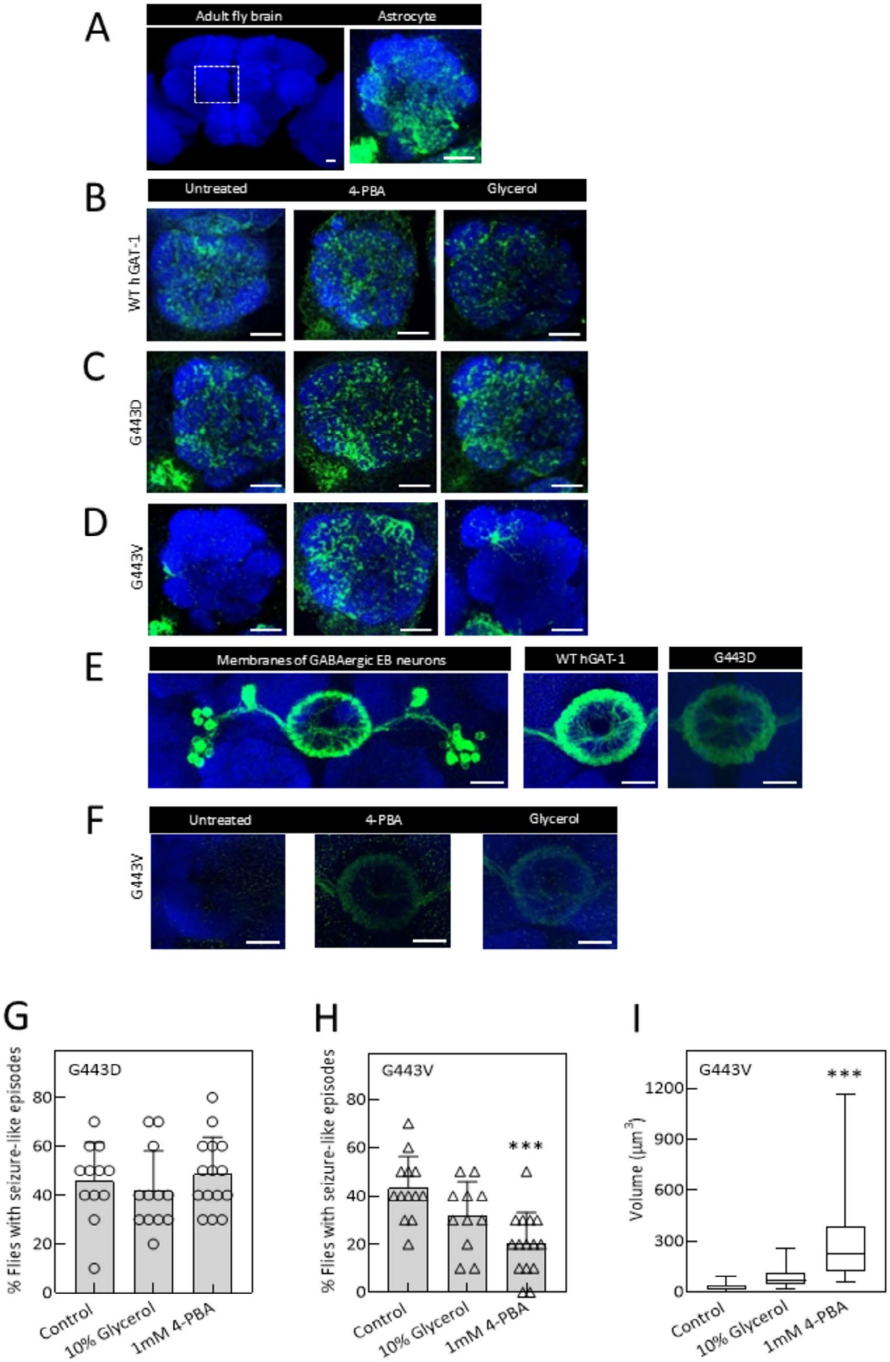
Pharmacological treatment ameliorates the neuronal and glial expression and activity of G443V mutant. (A) AL neuropile of adult fly brains is highlighted by the dotted box. Glial membrane (green), in a single section of AL, is labeled to denote the widespread distribution of astrocytic processes across the AL. (B to E) Flies of the said genotypes were either untreated (left panel) or treated with 4‐PBA (middle panel) or glycerol (right panel), as described in Methods. Protein expression was analyzed in a single section of AL neuropile (B to D) and ellipsoid body (E). (B) WT hGAT‐1 showed a wider expression in astrocytic processes and was unchanged in the treated flies. (C) G443D showed the expression pattern similar to the WT hGAT‐1 and glial membrane marker. (D) Untreated G443V mutant showed a restricted expression, mimicking the ER marker. Treatment with 4‐PBA restored the G443V expression in the glial processes. Glycerol had little impact on protein expression (H). (E) Presynaptic expression of G443V mutant was studied in the GABAergic EB neurons. We also studied the presynaptic expression of WT hGAT‐1 (middle panel) and G443D mutant (right panel). (F) Untreated mutant flies showed no presynaptic expression in the ellipsoid body. Treatment with both 4‐PBA and glycerol restored the presynaptic expression of the G443V mutant. (G) and (H) Heat‐induced seizures were studied after pharmacological treatment in G443D (G) and G443V (H) mutant flies. 4‐PBA treatment did not affect the seizure susceptibility in G443D flies (F). (G) 4‐PBA treatment significantly reduced the seizure activity in G443V flies. Glycerol treatment, although not statistically significant, showed slightly lower seizure activity in both mutants (G and H). (I) The AL expression of G443V after drug treatment was measured using Imaris 9.3 software in at least 10 adult brains of each condition. The statistical comparison was done by one‐way ANOVA, followed by Dunnett's test (***p < 0.001). Genotypes: (A) UAS‐ ALRM Gal4/+; UAS‐mCD8‐GFP/+; (B) ALRM Gal4; UAS‐YFP WT‐hGAT‐1; (C) ALRM Gal4; UAS‐YFP hGAT‐1‐G443D; (D) ALRM Gal4; UAS‐YFP hGAT‐1‐G443V; (E) Left panel; R20D01‐p65.AD/UAS mCD8‐GFP; R72B07‐GAL4.DBD/+; middle panel; R20D01‐p65.AD/+; UAS‐YFP WT hGAT‐1/R72B07‐GAL4.DBD; right panel; R20D01‐p65.AD/+; UAS‐YFP hGAT‐1‐G443D/R72B07‐GAL4.DBD. (F) R20D01‐p65.AD/+; UAS‐YFP hGAT‐1‐G443V/R72B07‐GAL4.DBD. Neuropiles are labeled by NCAD, in blue. Scale bar = 20 μm.

We next evaluated the impact of pharmacological treatment on GAT‐1 protein expression. Transgenic *Drosophila* lines expressing WT hGAT‐1, G443D, and G443V were given food supplemented with 1 mM 4‐PBA and 10% glycerol for 48 h. As shown in Figure 6A, protein expression was analyzed in a single plane of the AL. 4‐PBA and glycerol did not markedly alter the expression of WT GAT‐1 or G443D (Figure 6B,C, respectively). However, the expression of G443V was significantly enhanced by 4‐PBA, yielding an impressive 14‐fold increase in AL expression, while glycerol treatment had only a minor effect (Figure 6D,I). Additionally, we examined the effects of pharmacochaperoning on the neuronal expression of G443V. In GABAergic EB neurons (Figure 6E, left panel), both WT GAT‐1 and G443D (Figure 6, middle and right panel, respectively) indicated robust expression in the presynaptic region. Treatment with 4‐PBA and glycerol resulted in the presynaptic enrichment of G443V (Figure 6F). We also studied the effects of these compounds on seizure development in transgenic flies. The seizure phenotype of G443D flies stayed unaltered upon 4‐PBA exposure (Figure 6G). In contrast, 4‐PBA markedly reduced the seizure phenotype of G443V flies (Figure 6H), while glycerol treatment had a modest but statistically insignificant effect on both mutants (Figure 6G,H).

## 4 Discussion

The child harboring the hGAT‐1 mutation G443D experienced debilitating seizures, recurrent falls, and an inability to walk until the age of 20 months. In addition, she was afflicted with speech impairments and a diagnosis of autism spectrum disorder (ASD). The seizures remained refractory to conventional antiepileptic treatments, including ethosuximide and zonisamide, even at elevated doses. Although some symptomatic relief was observed with valproic acid and clobazam, these therapies also proved suboptimal. Despite a reduction in seizure intensity, the seizures persisted, consistent with the complex nature of managing this condition. Besides, the administration of valproic acid in young children warrants caution due to the associated risks [11].

The precise molecular mechanisms underlying *SLC6A1*‐associated pathologies are poorly understood. Previous studies, including our own, have demonstrated that disease‐associated GAT‐1 mutations result in a loss‐of‐function phenotype [10, 48]. Heterozygous GAT‐1 knockout (KO/+) mice exhibit approximately 70% of the GABA uptake activity observed in WT animals, yet show no overt phenotypic abnormalities [49]. In contrast, homozygous GAT‐1 KO mice display heightened sensitivity to pentylenetetrazole (PTZ)‐induced seizures, whereas heterozygous mice exhibit seizure thresholds comparable to WT [49]. Moreover, GAT‐1 KO mice present with absence seizures [49, 50]. Cope et al. reported that GAT‐1 KO mice exhibit increased tonic inhibition mediated by GABA_A_R receptors, particularly through activation of peri‐ and extrasynaptic receptors [50]. Thalamic GAT‐1 plays a critical role in regulating spike‐and‐wave discharges (SWDs), and enhanced extrasynaptic GABA_A_R activity in thalamocortical neurons is sufficient to induce absence seizures in murine models [50]. Notably, the δ subunit‐containing GABA_A_R is implicated in aberrant tonic inhibition [51]. In the absence of functional GAT‐1, synaptic GABA levels decline, diminishing phasic inhibition, while peri‐ and extrasynaptic GABA accumulates, enhancing tonic inhibition [50]. Given that patients with GAT‐1–associated disorders typically retain one functional allele, the pathophysiological mechanisms are, as yet, incompletely defined. In brief, it appears that neuronal hyperexcitability may arise not simply from impaired GABA clearance per se, but rather from the spatiotemporal dynamics of GABA signaling and the differential engagement of phasic versus tonic inhibition.

We here explored the molecular grounds underlying disease triggered by the epilepsy‐linked mutation G443D and a unique VUS, G443V, with the goal of identifying novel targets for more effective therapeutic strategies. Both G443D and ‐V were loss‐of‐function variants of hGAT‐1. It was plausible for the substitution of a small, flexible, and non‐polar glycine by a large, polar, and acidic aspartate to disturb transmembrane helix packing [11]. It was less foreseeable that its replacement by the slightly larger valine would yield equally, if not more, deleterious consequences. G443 is strikingly conserved across the SLC6 transporter family (Table 1), underscoring its critical role in governing the expression, structure, and function of these proteins. Mutations at the analogous glycine residues in other human SLC6 transporters have been classified as pathogenic or likely deleterious, thereby conspicuously highlighting the significance of this particular glycine (Table 3): for example, G466R in hCRT‐1 leads to the creatine transporter deficiency (CTD) syndrome [52], which is also manifested by epilepsy and severe intellectual disability.

**TABLE 3:** G443‐hGAT1‐equivalent mutations in other SLC6 transporters, with the corresponding pathogenic predictions and their alpha missense scores.

Gene	Name	Variants	Predictions	Missense score	Uniprot ID
*slc6a1*	hGAT1	G443D	Likely pathogenic (MAE)	1	RCV003763760
		G443V	Likely pathogenic	1	RCV001267259
*slc6a2*	hNET	G465E	Probably damaging, Probably deleterious	1	COSV54915043
		G465R		0.99	rs1297555256
*slc6a3*	hDAT	G468S	Deleterious, likely pathogenic	0.93	rs1579714321
*slc6a4*	hSERT	G485E	Probably deleterious, pathogenic	0.99	COSV55565385
*slc6a6*	hTAUT	G451A	Probably deleterious, pathogenic	0.98	rs780841499
*slc6a8*	hCRT1	G466R	Highly likely deleterious, pathogenic (CTD)	1	RCV000990970
		G466V	Probably deleterious, pathogenic (CTD)	1	COSV53472199
*slc6a9*	hGlyT1	G520R	Probably deleterious, pathogenic	1	rs201598031
*slc6a12*	hBGT1	G444R	Probably deleterious, pathogenic	0.99	rs1011747088
		G444W		1	COSV62886160
*slc6a13*	hGAT2	G439R	Probably deleterious, pathogenic	0.99	COSV58257496
		G439V	Quite likely deleterious, pathogenic	0.99	rs779416804
*slc6a14*	hATB^0+^	G470E	Probably deleterious, pathogenic	0.99	COSV64146261
*slc6a17*	hNTT4	G516R	Highly likely deleterious, pathogenic	1	COSV58992223
		G516* (stop)	Highly likely deleterious	—	COSV58992223
*slc6a18*	hB^0^AT3	G464E	Probably deleterious, pathogenic	0.44	COSV99722344
		G464R		0.32	rs568858657
*slc6a19*	hB^0^AT1	G478S	Probably deleterious, pathogenic (VUS)	0.48	RCV001915499
*slc6a20*	hXTRP3	G453R	Probably deleterious, pathogenic	0.97	rs867489501

The impact of glycine residues on the structure–function dynamics of SLC6 transporters is well‐established. For instance, glycines flanking the highly conserved GXXXRXG motif of the human norepinephrine transporter (NET) are essential for its proper expression and functional integrity [53]. Similarly, glycines within the glycophorin A motif, GXXXG, are essential for dimer formation and inserting hSERT into the plasma membrane via helix–helix interactions in the lipid bilayer [54]. Furthermore, a folding‐deficient *Drosophila* DAT mutant, G108Q, induces a sleepless phenotype in flies, resulting from protein misfolding ^14^. G108 is part of a GXXXG‐related motif, which stabilizes the interaction between TMs 3 and 12. Disruption of this motif interferes with the proper packing of helix 12, thereby perturbing the overall structure [34]. The G108Q mutant was responsive to the pharmacochaperone action of noribogaine and to the HSP70 blocker pifithrin‐μ, to an extent that reinstated normal sleep duration in flies carrying this mutation [14]. The pathogenic G132V mutation at the corresponding residue in hCRT‐1 gives rise to CTD in affected boys, a consequence of profound protein folding deficiency [40]. This paradigm is hardly astonishing: many glycine residues reside in hydrophobic TM helices of membrane proteins and are predominantly involved in helix–helix interactions and support their packing by avoiding contact with the lipid bilayer [55]. Glycines customarily reduce protein aggregation, due to their low propensity to form β‐strands [56]. The exchange of G443 by valine and aspartate elicited distinctive effects on hGAT‐1 expression. The valine substitution proved more unfavorable, which was surprising given the nature of their side chains, as described above. It is tempting to speculate that the negatively charged aspartate (in G443D) is involved in electrostatic and hydrogen bond interactions, possibly forming salt bridges with basic amino groups, thereby contributing to the overall stability of the protein structure [57, 58]. Aspartates are key interaction partners of cationic residues, like arginine, which dictate the pH‐low insertion peptide (pHLIP)–membrane equilibria in transmembrane peptides [59]. During P2X purinoreceptor biosynthesis, for instance, aspartates quench the hydrogen bonding groups in the membrane, to drive local oligomer formation and increase the concentration of homotrimers [60]. This further hints at aspartate residues acting as a scaffold for proper positioning and orientation of recognition protein surfaces in the ectodomain. Besides their ability to promote oligomerization in lipid bilayers, aspartates also foster the incorporation of hydrophobic transmembrane helices via the Sec61 translocon [61]. Some of the above may rationalize why G443D is targeted to the plasma membrane, contrasting the effects on GAT‐1 protein expression, elicited by the more conservative mutation to valine (G443V). G443D, being expressed at the cell surface, but non‐functional, implies that it impairs GABA binding and/or translocation via the hGAT‐1. In G443V, the isopropyl side chain likely disrupts the folding process and hinders ER export, as previously established for numerous glycine to valine mutations in the P‐glycoprotein [62] and the A288V variant in hGAT‐1 [1].

We identified two small molecules demonstrating rescue potential on G443V and D mutants, in vitro and in vivo. G443V was restored by 4‐PBA, a molecule with manifold mechanisms of action. For instance, 4‐PBA was reported to extend the lifespan in flies due to increased histone acetylation [63]. In our hands, it lived up to its repute as a chemical chaperone, efficiently correcting misfolded CRT‐1 and GAT‐1 mutants [1, 40]. 4‐PBA also rescued two clinically relevant, trafficking‐deficient, GlyT2 (SLC6A5) variants [64]. Its therapeutic potential is immense: glycerol phenylbutyrate (Ravicti) is currently used in a clinical trial in patients afflicted with SLC6A1‐ (i.e., GAT‐1) and syntaxin‐binding protein 1 (STXBP1)‐linked developmental and epileptic encephalopathy [65]. The beneficial effects of 4‐PBA are pervasive and presumably synergistic. It prevents protein aggregation and averts ER stress [66] and is known to decline HSC70 protein expression (by reducing HSC70 mRNA stability, but not the synthesis thereof) [67]. Importantly, 4‐PBA was successfully used in amyotrophic lateral sclerosis (ALS) patients, whose lifespan was appreciably extended by 4‐PBA treatment [68, 69].

The partially folded G443D variant was amenable to functional rescue by glycerol. The chaperoning effects of glycerol were previously observed for ER‐trapped cystic fibrosis variants of the CFTR [39]. It also prevented the formation of protein aggregates of misfolded disease mutants of the membrane‐associated guanylate kinase (MAGUK), linked to intellectual disability in boys [70]. In addition, aquaporin‐2 mutants associated with nephrogenic diabetes insipidus correctly translocated to the membrane following glycerol treatment [71]. Glycerol is thought to improve protein stability owing to its amphiphilic properties, which allow it to act as an interface between aggregation‐prone hydrophobic moieties and the polar solvent [72].

## 5 Conclusion

The identification of small molecules as potential therapeutic agents offers promising avenues for rescuing loss‐of‐function and/or misfolded pathogenic variants in neurotransmitter transporters. These findings not only advance our understanding of the molecular mechanisms of disease, but pave the way for further clinical exploration and point toward enhanced treatment strategies for severe neurological disorders, such as for example, hGAT‐1‐related epilepsy and intellectual disability.

## Author Contributions

N.S.: investigation; methodology; formal analysis; writing – original draft. V.K.: investigation; methodology; formal analysis. R.Z.: investigation; formal analysis; methodology; writing – original draft. K.C.: investigation. A.B.: methodology; writing – review and editing. T.H.: methodology; formal analysis. H.H.S.: funding acquisition; methodology. A.S.K.: investigation; methodology; formal analysis; writing – original draft; writing – review and editing. S.S.: conceptualization; funding acquisition; writing – review and editing; writing – original draft; formal analysis; project administration.

## Disclosure

The authors R.Z., K.C., and A.B. are employed by Nanion Technologies GmbH, which provides the SSME device SURFE^2^R N1.

## Conflicts of Interest

The authors declare no conflicts of interest.

## Supporting information


Figure S1.

Figure S2.

Figure S3.

Figure S4.

## Data Availability

The data that support the findings of this study are available from the corresponding authors, upon reasonable request.
