# Supplementary figures and images for: Rescue of Epilepsy‐Associated Mutations of the Highly Conserved Glycine Residue 443 in the Human GABA Transporter 1
Source: FASEB J. 2025 Jun 9;39(11):e70614. doi: 10.1096/fj.202403159RR (PMC12146831; doi:10.1096/fj.202403159RR)

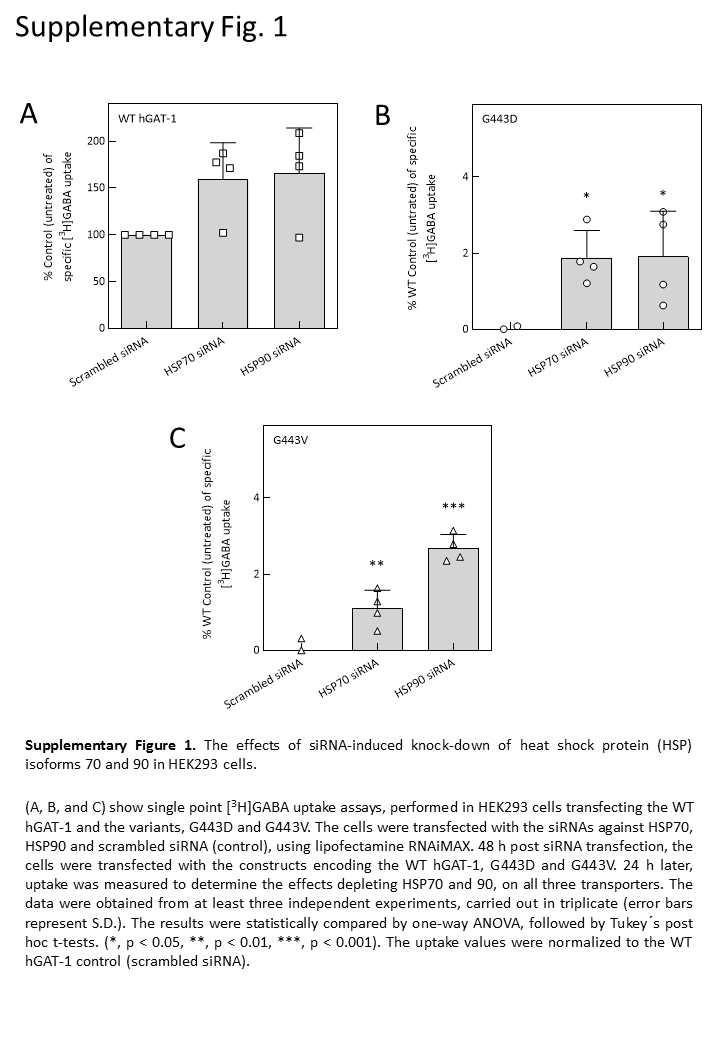

Supplement: Supplementary file 1 — Figure S1. [file FSB2-39-e70614-s004.tif]

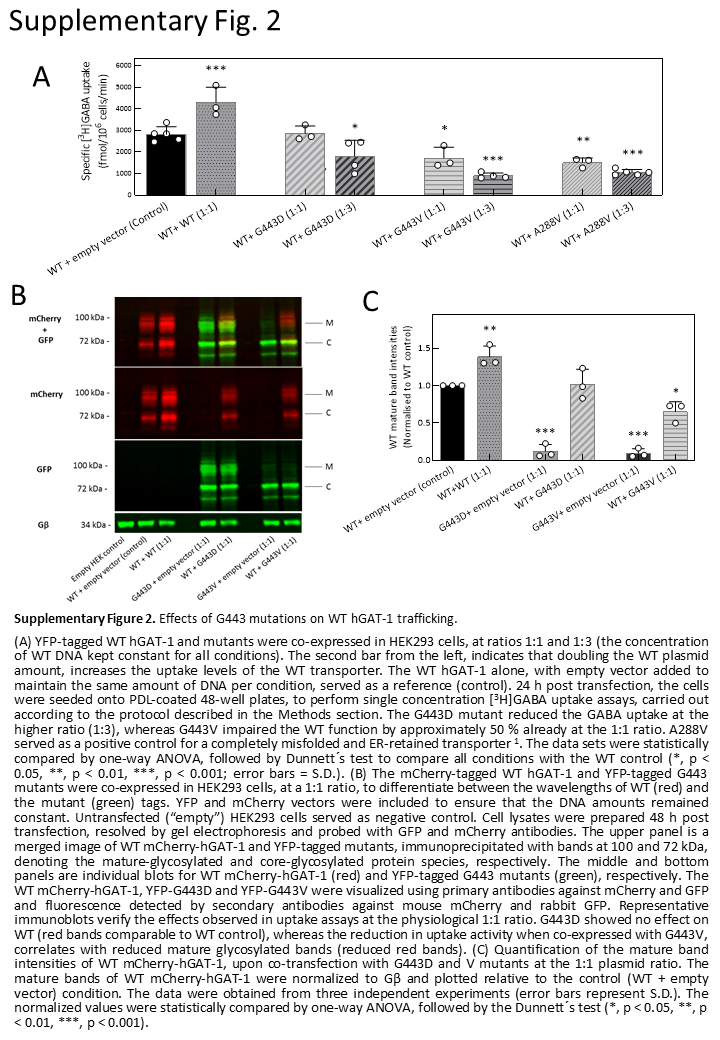

Supplement: Supplementary file 2 — Figure S2. [file FSB2-39-e70614-s001.tif]

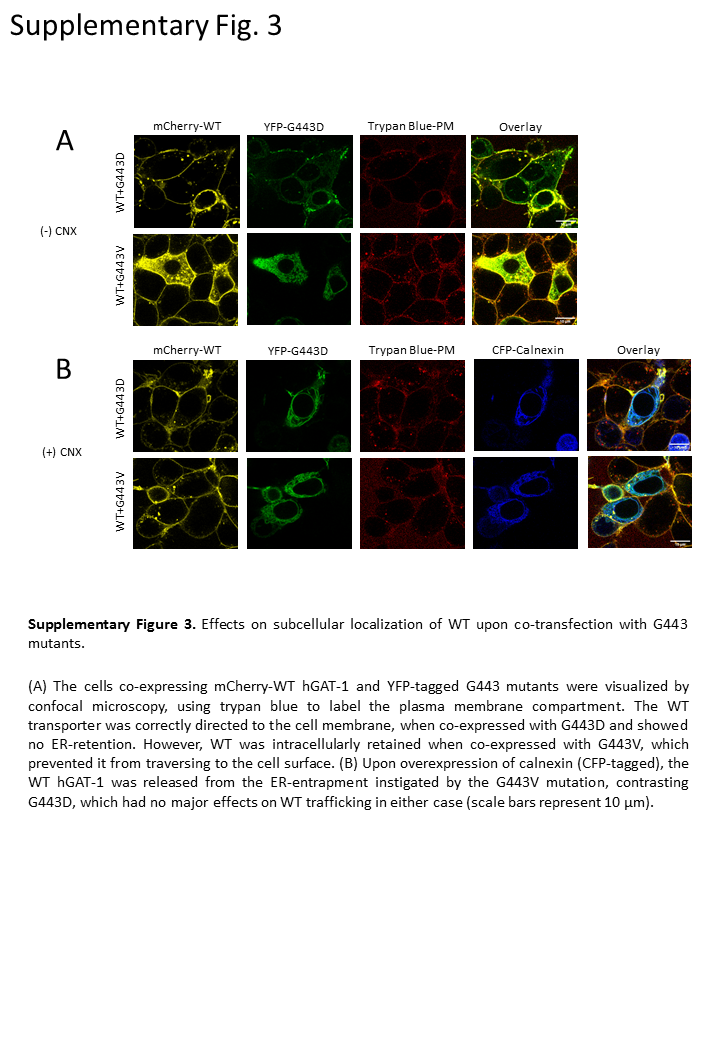

Supplement: Supplementary file 3 — Figure S3. [file FSB2-39-e70614-s003.tif]

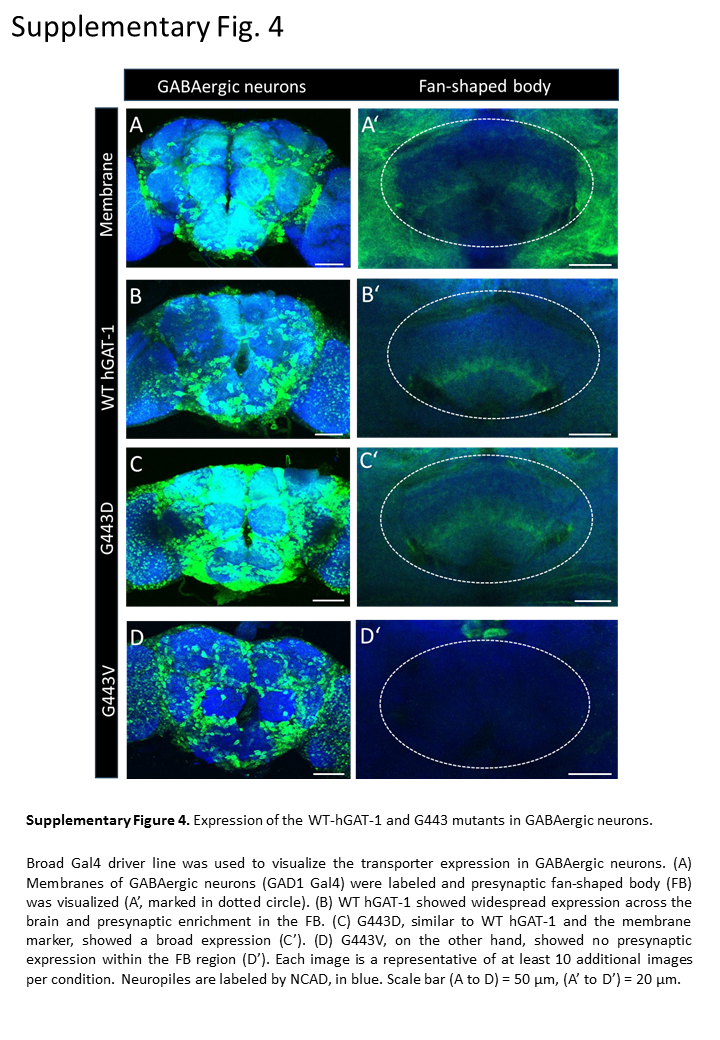

Supplement: Supplementary file 4 — Figure S4. [file FSB2-39-e70614-s002.tif]
